# Trends and efficacy of omitting axillary lymph node dissection in early‐stage male breast cancer with limited nodal involvement: A population‐based cohort study

**DOI:** 10.1002/cam4.70243

**Published:** 2024-10-08

**Authors:** Hanzhao Yang, Yuxia Ruan, Jiabin Wang, Jianghua Qiao, Zhenzhen Liu

**Affiliations:** ^1^ Department of Breast Disease, Henan Breast Cancer Center The Affiliated Cancer Hospital of Zhengzhou University and Henan Cancer Hospital Zhengzhou China

**Keywords:** male breast cancer, SEER, sentinel lymph node biopsy, survival

## Abstract

**Background:**

The effectiveness of sentinel lymph node biopsy (SLNB) versus axillary lymph node dissection (ALND) in managing early‐stage male breast cancer (MBC) patients with T1‐2 tumors and limited lymph node metastasis, all receiving radiotherapy, remains uncertain. This study examines trends and survival outcomes for SLNB and ALND in the United States.

**Methods:**

We conducted a retrospective analysis of the Surveillance, Epidemiology, and End Results (SEER) data from 2010 to 2020 for MBC patients with T1‐2 tumors and 1–2 positive lymph nodes undergoing radiotherapy. Patients were classified by nodes removed (SLNB ≤5, ALND ≥10), comparing overall survival (OS) and breast cancer‐specific survival (BCSS) between the groups before and after propensity score matching.

**Results:**

Of 299 MBC patients analyzed, SLNB usage increased from 18.8% in 2010 to 61.0% in 2020. Multivariable logistic regression highlighted significant associations of SLNB use with diagnosis year, race, surgery type, positive lymph node count, and metastasis size. No significant differences in 5‐year OS (77.98% SLNB vs. 85.85% ALND, *p* = 0.337) or BCSS (91.54% SLNB vs. 94.97% ALND, *p* = 0.214) were observed. Propensity score matching (96 patients per group) confirmed similar 5‐year OS (83.9% for SLNB vs. 82.0% for ALND, *p* = 0.925) and BCSS (90.1% for SLNB vs. 96.9% for ALND, *p* = 0.167).

**Conclusion:**

SLNB and ALND provide comparable survival outcomes in early‐stage MBC patients with limited lymph node metastasis undergoing radiotherapy. The increased utilization of SLNB supports its consideration to reduce surgical morbidity in selected MBC patients despite limited direct evidence.

## 1 INTRODUCTION

Male breast cancer (MBC) represents a rare subset of breast cancer, comprising less than 1% of all cases,^1^ and poses unique challenges in clinical management due to its rarity. The scarcity of MBC has resulted in treatment guidelines that predominantly extrapolate from female breast cancer (FBC) research and clinical experience, despite notable differences in biological characteristics and clinical presentation between genders. This extrapolation is primarily due to the challenges associated with conducting large‐scale clinical trials specifically for MBC,^2^ which has led to a significant gap in evidence‐based treatment strategies tailored for male patients.

Sentinel lymph node biopsy (SLNB) has emerged as a less invasive surgical approach that mitigates the risk of complications^3^, ^4^ such as lymphedema, numbness, and pain, commonly associated with axillary lymph node dissection (ALND) in the management of early‐stage breast cancer. Pivotal studies, including the ACOSOG Z0011^5^ and EORTC AMAROS^6^ trials, have demonstrated that for early‐stage FBC patients (cT1‐2 with postoperative radiotherapy to the breast and/or the region) with one or two positive sentinel lymph nodes, SLNB alone yields survival rates comparable to those of ALND, leading to a substantial decline in ALND utilization over the past decade.^7^, ^8^ However, the direct application of these findings to MBC is cautioned due to differences in disease presentation, such as more advanced stage and a higher incidence of axillary lymph node metastasis in male patients.^1^

Notwithstanding the paucity of research on the feasibility of sparing ALND in early‐stage MBC patients with limited sentinel lymph node metastasis, existing studies, such as the one by Chung et al.,^9^ suggest potential disparities in overall survival (OS) between MBC patients undergoing SLNB and those receiving ALND. This discrepancy may partly result from variations in postoperative care, notably the absence of radiotherapy in a significant proportion of the study cohort, diverging from the protocols employed in the Z0011^5^ and AMAROS trials.^6^

The epidemiology of omitting ALND in early‐stage MBC patients with limited sentinel lymph node positivity in the real world remains unclear since the landmark trials. Additionally, the optimal axillary treatment for these patients is uncertain. By extracting data from the Surveillance, Epidemiology, and End Results (SEER) database, our study aimed to investigate the utilization trends of SLNB among MBC patients in the United States with T1‐2 staging and 1–2 lymph node metastases who underwent radiotherapy. We intend to compare the survival outcomes—OS and breast cancer‐specific survival (BCSS)—between SLNB and ALND treatments.

## 2 MATERIALS AND METHODS

### 2.1 Data sources

This retrospective cohort study utilized data extracted from the SEER 17 registries database, as of the release date of January 18, 2024, employing the National Cancer Institute's SEER*Stat software (version 8.4.3). The SEER database, capturing approximately 28% of the US population, was used to identify cases of MBC diagnosed between 2010 and 2020.

### 2.2 Study population and selection criteria

We included a cohort of 7278 male patients diagnosed with primary breast cancer, identified through specific International Classification of Diseases for Oncology, Third Edition (ICD‐O‐3) histology codes: 8500 for infiltrating ductal carcinoma and 8520 for lobular carcinoma. Our selection criteria focused on patients with clinical stage T1 or T2 tumors and 1–2 positive axillary lymph nodes. Surgical treatment data were categorized based on procedural codes for breast‐conserving surgery (20‐24) and mastectomy (30, 40–76, 80), with the inclusion of adjuvant radiotherapy when applicable.

**FIGURE 1:**
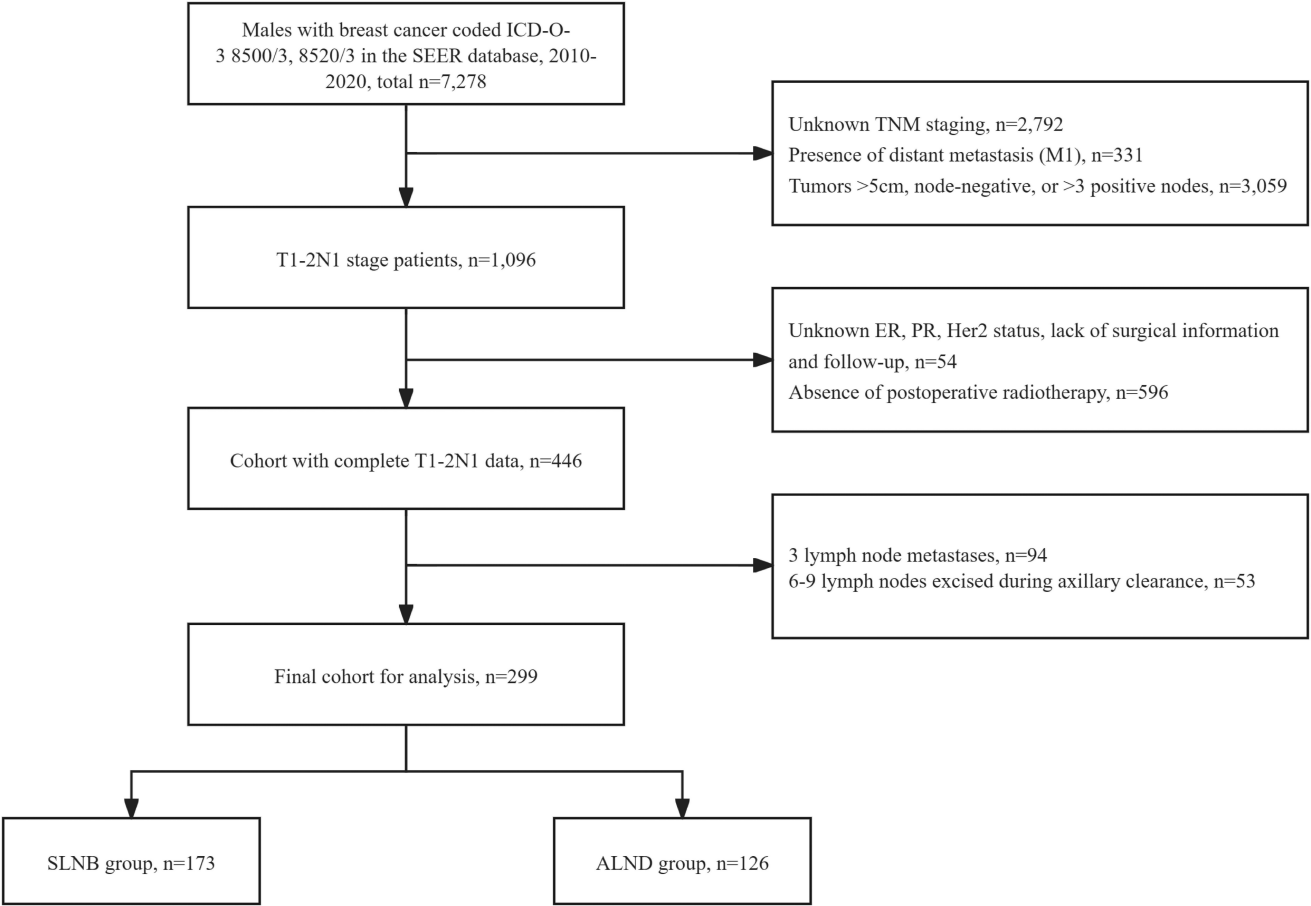
Cohort selection flowchart.

Exclusion criteria were applied to omit patients with distant metastasis, unknown estrogen receptor (ER) /progesterone receptor (PR) or Her‐2 status, or incomplete data regarding vital status or survival time. The determination of the axillary surgical approach—ALND or SLNB—was inferred from the recorded number of lymph nodes removed: ALND was presumed for patients with ≥10 nodes excised, whereas SLNB was considered for cases with ≤5 nodes removed. This classification was based on established guideline recommendations^10^ and prior literature^11^, ^12^ (Figure 1). The Institutional Review Board of The Affiliated Cancer Hospital of Zhengzhou University & Henan Cancer Hospital deemed this research exempt from review and waived the requirement for informed consent, as the analysis involved publicly available, de‐identified patient data.

### 2.3 Statistical analysis

We summarized the baseline characteristics of the study population using descriptive statistics. Continuous variables were presented as means ± standard deviations for normally distributed data, and categorical variables were reported as counts and percentages. Differences between groups for continuous variables were assessed using independent samples *t*‐tests for normally distributed variables and the Mann–Whitney *U* test for non‐normally distributed variables. Chi‐squared tests or Fisher's exact tests were employed for categorical variables, depending on the appropriateness for the data distribution. To investigate the temporal trends in the adoption of SLNB among MBC patients from 2010 to 2020, we analyzed annual counts of patients undergoing ALND and SLNB. The Cochran–Armitage trend test was applied to assess the significance of these trends. For evaluating the association of study variables with the outcome of SLNB, univariate and multivariate logistic regression models were implemented. Variables included in the multivariate model were those with a univariate association with any SLNB at a *p*‐value less than 0.10. The Kaplan–Meier method was utilized to estimate 5‐year OS and BCSS, with the log‐rank test comparing survival outcomes between groups. Survival time was calculated from the date of diagnosis to the date of death or last known contact, with a maximum follow‐up of 5 years. Cox proportional hazards models were used to estimate hazard ratios (HRs) and 95% confidence intervals (CIs) for survival comparisons, ensuring the proportional hazards assumption was verified using Schoenfeld residuals.

To adjust for potential confounders and selection bias, propensity score matching (PSM) was performed. Covariates significant in group comparisons (*p* < 0.05) were included in a logistic regression model to generate propensity scores. Matching was conducted on a 1:1 basis using nearest‐neighbor matching without replacement, with a caliper width set at 0.2 standard deviations of the logit of the propensity score. Baseline characteristics were re‐evaluated post‐matching to ensure balance between groups, and survival analyses for OS and BCSS were replicated on the matched groups. A sensitivity analysis was conducted to address any potential bias related to the definition of SLNB, particularly in light of the Z0011 trial's median sentinel lymph node count.^5^ We redefined SLNB as the dissection of 1–4 lymph nodes and performed stratified sensitivity analyses accordingly. All analyses were executed using the R software (version 4.3.1, R Foundation for Statistical Computing, Vienna, Austria). Statistical significance was determined by a two‐sided *p*‐value of less than 0.05.

## 3 RESULTS

### 3.1 Baseline characteristics of the cohort

Our cohort consisted of 299 MBC patients, stratified into ALND (126 patients) and SLNB (173 patients) groups. The majority were White (80.3%) and married (65.9%), with infiltrating ductal carcinoma being the most common histology (97.0%). The mean age was 64.8 ± 11.2 years. Patients in the SLNB group were significantly older than those in the ALND group (*p* = 0.027). The SLNB group also had a higher rate of breast‐conserving surgery (20.8% vs. 6.3%, *p* < 0.001) and single sentinel lymph node metastasis (80.3% vs. 54.0%, *p* < 0.001), but a lower rate of chemotherapy (55.5% vs. 69.0%, *p* = 0.018). No significant differences were observed in tumor size and molecular subtype between the groups (Table 1).

**TABLE 1:** Patient demographics and baseline characteristics.

Characteristic	Total (*n* = 299)	Lymph node dissection group		*p*‐value
		SLNB (*n* = 173)	ALND (*n* = 126)	
Year of diagnosis				0.088
2010	16 (5.4%)	3 (1.7%)	13 (10.3%)	
2011	11 (3.7%)	4 (2.3%)	7 (5.6%)	
2012	29 (9.7%)	18 (10.4%)	11 (8.7%)	
2013	19 (6.4%)	13 (7.5%)	6 (4.8%)	
2014	23 (7.7%)	14 (8.1%)	9 (7.1%)	
2015	23 (7.7%)	15 (8.7%)	8 (6.3%)	
2016	36 (12.0%)	19 (11.0%)	17 (13.5%)	
2017	27 (9.0%)	17 (9.8%)	10 (7.9%)	
2018	37 (12.4%)	21 (12.1%)	16 (12.7%)	
2019	37 (12.4%)	24 (13.9%)	13 (10.3%)	
2020	41 (13.7%)	25 (14.5%)	16 (12.7%)	
Age, Mean ± SD	64.8 ± 11.2	65.9 ± 11.0	63.1 ± 11.2	0.027
Age group				0.159
1	89 (29.8%)	46 (26.6%)	43 (34.1%)	
2	210 (70.2%)	127 (73.4%)	83 (65.9%)	
Marital status				0.802
Married	197 (65.9%)	115 (66.5%)	82 (65.1%)	
Unmarried	102 (34.1%)	58 (33.5%)	44 (34.9%)	
Race				0.100
White	240 (80.3%)	146 (84.4%)	94 (74.6%)	
Black	42 (14.0%)	17 (9.8%)	25 (19.8%)	
Others	12 (4.0%)	7 (4.0%)	5 (4.0%)	
Unknown	5 (1.7%)	3 (1.7%)	2 (1.6%)	
Histological type				>0.999
Ductal	290 (97.0%)	168 (97.1%)	122 (96.8%)	
Lobular	9 (3.0%)	5 (2.9%)	4 (3.2%)	
Grade				0.822
1	13 (4.4%)	8 (4.6%)	5 (4.0%)	
2	87 (29.1%)	47 (27.2%)	40 (31.7%)	
3	73 (24.4%)	42 (24.3%)	31 (24.6%)	
Unknown	126 (42.1%)	76 (43.9%)	50 (39.7%)	
Clinical T stage				0.362
1	123 (41.1%)	75 (43.4%)	48 (38.1%)	
2	176 (58.9%)	98 (56.6%)	78 (61.9%)	
ER‐receptor status				0.699
Positive	293 (98.0%)	170 (98.3%)	123 (97.6%)	
Negative	6 (2.0%)	3 (1.7%)	3 (2.4%)	
PR‐receptor status				0.748
Positive	271 (90.6%)	156 (90.2%)	115 (91.3%)	
Negative	28 (9.4%)	17 (9.8%)	11 (8.7%)	
HER2‐receptor status				0.981
Positive	31 (10.4%)	18 (10.4%)	13 (10.3%)	
Negative	268 (89.6%)	155 (89.6%)	113 (89.7%)	
Breast surgery				<0.001
Mastectomy	255 (85.3%)	137 (79.2%)	118 (93.7%)	
BCS	44 (14.7%)	36 (20.8%)	8 (6.3%)	
Number of positive lymph nodes				<0.001
1	207 (69.2%)	139 (80.3%)	68 (54.0%)	
2	92 (30.8%)	34 (19.7%)	58 (46.0%)	
Sentinel lymph node met size				<0.001
Micrometastases (2 mm)	64 (21.4%)	53 (30.6%)	11 (8.7%)	
Macrometastases (>2 mm)	235 (78.6%)	120 (69.4%)	115 (91.3%)	
Chemotherapy				0.018
Yes	183 (61.2%)	96 (55.5%)	87 (69.0%)	
No/Unknown	116 (38.8%)	77 (44.5%)	39 (31.0%)	

Abbreviations: ALND, axillary lymph node dissection; BCS, breast‐conserving therapy; HER2, human epidermal growth factor receptor 2; HR, hormone receptor; PR, progesterone receptor; SD, standard deviation; SLNB, sentinel lymph node biopsy.

### 3.2 Trends in sentinel lymph node biopsies in male breast cancer in the United States

**FIGURE 2:**
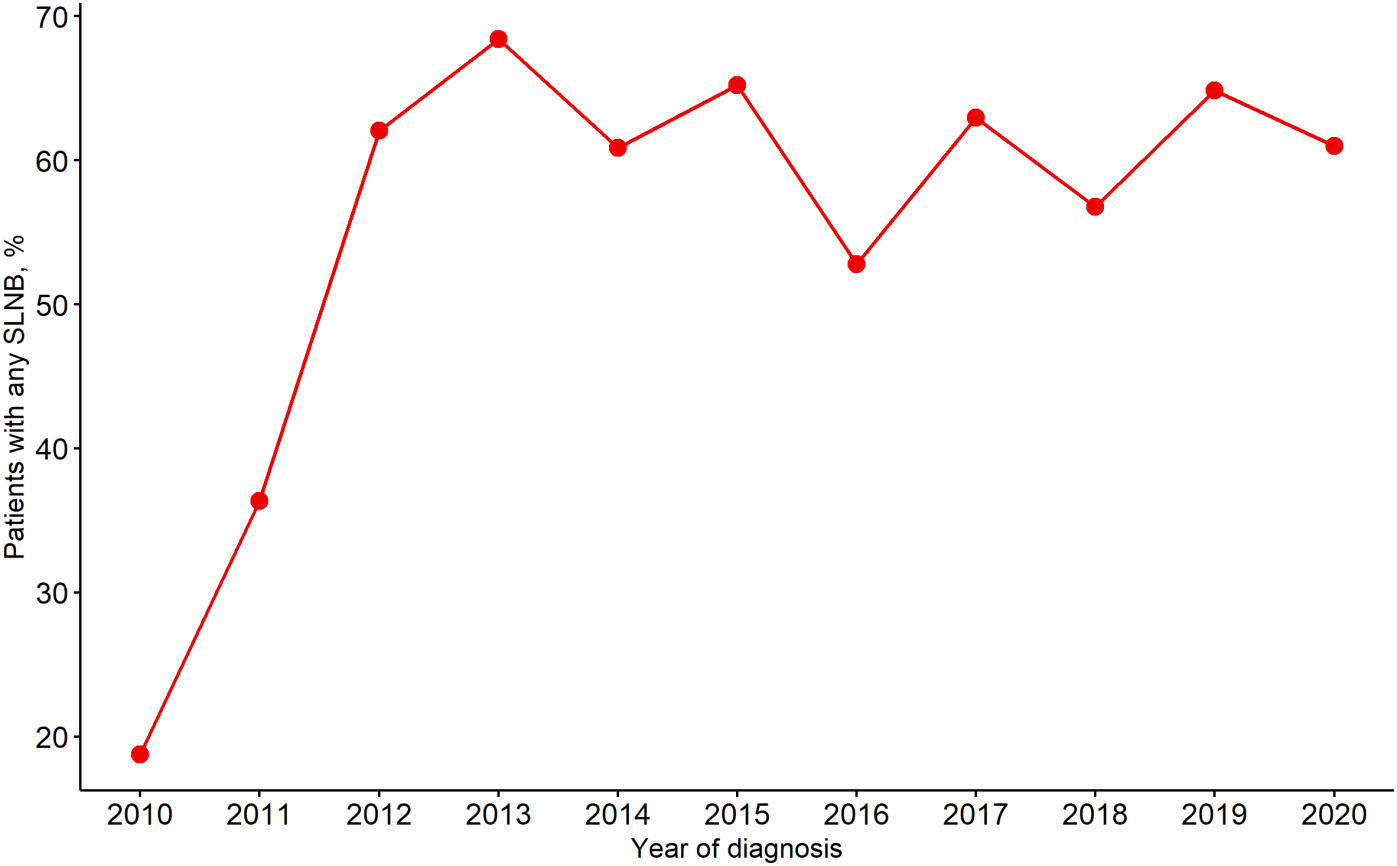
Trends in axillary lymph node dissection omission in male breast cancer: 2010–2020.

The utilization rate of SLNB increased from 18.8% in 2010 to 61.0% in 2020, with a significant rise observed during 2010–2012. The Cochran–Armitage trend test indicated a statistically significant increase in the early period (*p* < 0.05 for 2010–2012), but this trend plateaued from 2012 to 2020. Linear regression analysis confirmed a positive correlation between the year of diagnosis and SLNB usage, with a scatterplot illustrating an initial surge followed by stabilization (Figure 2). Multivariable logistic regression analysis revealed that the use of SLNB was significantly associated with the year of diagnosis (2020 vs. 2010; OR, 6.06; 95% CI, 1.39–34.05), race (Black vs. White; OR, 0.43; 95% CI, 0.19–0.96), type of breast surgery (BCS vs. mastectomy; OR, 5.11; 95% CI, 2.06–14.35), number of positive lymph nodes (2 vs. 1; OR, 0.38; 95% CI, 0.21–0.67), and size of sentinel lymph node metastasis (macrometastases vs. micrometastases; OR, 0.29; 95% CI, 0.13–0.61) (Table 2).

**TABLE 2:** Univariate and multivariate logistic regression for odds of undergoing sentinel lymph node biopsy in male breast cancer patients.

Characteristic	OR (95% CI)	*p*‐value	OR (95% CI)	*p*‐value
Year of diagnosis				
2010	1 [Reference]		1 [Reference]	
2011	2.48 (0.43–15.86)	0.312	1.68 (0.23–13.23)	0.606
2012	7.09 (1.81–36.32)	0.009	6.56 (1.40–39.34)	0.024
2013	9.39 (2.12–53.84)	0.006	14.81 (2.79–101.59)	0.003
2014	6.74 (1.63–35.91)	0.013	7.26 (1.44–46.55)	0.023
2015	8.13 (1.95–43.82)	0.007	7.44 (1.47–47.70)	0.022
2016	4.84 (1.30–23.83)	0.029	5.28 (1.19–30.31)	0.040
2017	7.37 (1.85–38.26)	0.008	5.76 (1.21–34.77)	0.037
2018	5.69 (1.53–27.96)	0.016	6.15 (1.38–35.67)	0.026
2019	8.00 (2.13–39.76)	0.004	8.19 (1.83–47.70)	0.010
2020	6.77 (1.84–33.04)	0.008	6.06 (1.39–34.05)	0.024
Age	1.02 (1.00–1.05)	0.028	1.02 (0.99–1.05)	0.122
Age group				
1	1 [Reference]			
2	1.43 (0.87–2.36)	0.160		
Marital status				
Married	1 [Reference]			
Unmarried	0.94 (0.58–1.53)	0.802		
Race				
White	1 [Reference]		1 [Reference]	
Black	0.44 (0.22–0.85)	0.015	0.43 (0.19–0.96)	0.043
Others	0.90 (0.28–3.12)	0.863	1.12 (0.27–4.72)	0.873
Unknown	0.97 (0.16–7.43)	0.970	0.85 (0.09–8.69)	0.881
Histological type				
Ductal	1 [Reference]			
Lobular	0.91 (0.24–3.73)	0.887		
Grade				
1	1 [Reference]			
2	0.73 (0.21–2.38)	0.612		
3	0.85 (0.24–2.79)	0.788		
Unknown	0.95 (0.27–3.01)	0.932		
Clinical T stage				
1	1 [Reference]			
2	0.80 (0.50–1.28)	0.362		
ER‐receptor status				
Positive	1 [Reference]			
Negative	0.72 (0.13–3.97)	0.695		
PR‐receptor status				
Positive	1 [Reference]			
Negative	1.14 (0.52–2.59)	0.748		
HER2‐receptor status				
Positive	1 [Reference]			
Negative	0.99 (0.46–2.09)	0.981		
Breast surgery				
Mastectomy	1 [Reference]		1 [Reference]	
BCS	3.88 (1.82–9.27)	<0.001	5.11 (2.06–14.35)	<0.001
Number of positive lymph nodes				
1	1 [Reference]		1 [Reference]	
2	0.29 (0.17–0.48)	<0.001	0.38 (0.21–0.67)	<0.001
Sentinel lymph node met size				
Micrometastases (2 mm)	1 [Reference]		1 [Reference]	
Macrometastases (>2 mm)	0.22 (0.10–0.42)	<0.001	0.29 (0.13–0.61)	0.002
Chemotherapy				
Yes	1 [Reference]		1 [Reference]	
No/unknown	1.79 (1.11–2.91)	0.018	1.09 (0.57–2.07)	0.801

Abbreviations: BCS, breast‐conserving therapy; CI, confidence interval, HER2, human epidermal growth factor receptor 2; HR, hormone receptor, PR, progesterone receptor; OR, odds ratio.

### 3.3 Survival analysis

**FIGURE 3:**
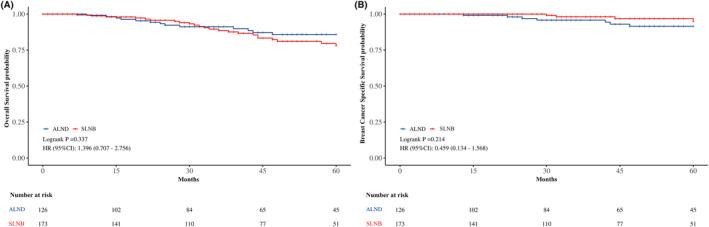
Kaplan–Meier survival curves of the patients. (A) 5‐year OS between SLNB and ALND groups before PSM; (B) 5‐year BCSS between SLNB and ALND groups before PSM; ALND, axillary lymph node dissection; BCSS, breast cancer specific survival; OS, overall survival; PSM, propensity score matching; SLNB, sentinel lymph node biopsy.

The median follow‐up was 50.0 months for the entire cohort, with the SLNB group at 47.0 months and the ALND group at 52.0 months, the Kaplan–Meier analysis pre‐propensity score matching revealed no significant difference in 5‐year OS between the SLNB and ALND groups (77.98% vs. 85.85%, respectively; HR for SLNB vs. ALND: 1.396, 95% CI: 0.707–2.756, *p* = 0.337) nor in 5‐year BCSS (91.54% vs. 94.97%, respectively; HR for SLNB vs. ALND: 0.459, 95% CI: 0.134–1.568, *p* = 0.214) (Figure 3).

### 3.4 Post‐propensity score matching outcomes

**FIGURE 4:**
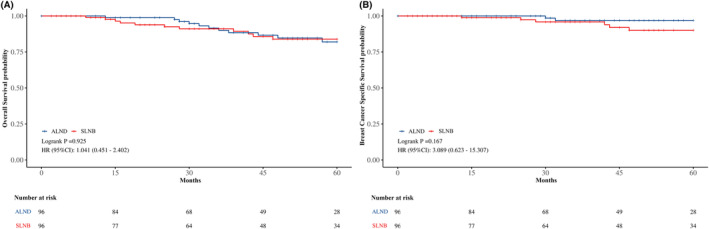
Kaplan–Meier survival curves of the patients. (A) 5‐year OS between SLNB and ALND groups after PSM; (B) 5‐year BCSS between SLNB and ALND groups after PSM; ALND, axillary lymph node dissection; BCSS, breast cancer specific survival; OS, overall survival; PSM, propensity score matching; SLNB, sentinel lymph node biopsy.

After propensity score matching, the groups were well‐balanced, each comprising 96 patients (Table 3). The matched analysis revealed similar 5‐year OS rates (SLNB: 83.9% vs. ALND: 82.0%; HR for SLNB vs. ALND: 1.041, 95% CI: 0.451–2.402, *p* = 0.925) and 5‐year BCSS rates (SLNB: 90.1% vs. ALND: 96.9%; HR for SLNB vs. ALND: 3.089, 95% CI: 0.623–15.307, *p* = 0.167) (Figure 4).

**TABLE 3:** Patient demographics and baseline characteristics after propensity score matching.

Characteristic	Total (*n* = 192)	Lymph node dissection group		*p*‐value
		SLNB (*n* = 96)	ALND (*n* = 96)	
Age, Mean ± SD	64.8 ± 10.6	65.3 ± 10.7	64.4 ± 10.6	0.583
Age group				0.751
1	56 (29.2%)	27 (28.1%)	29 (30.2%)	
2	136 (70.8%)	69 (71.9%)	67 (69.8%)	
Marital status				0.371
Married	120 (62.5%)	57 (59.4%)	63 (65.6%)	
Unmarried	72 (37.5%)	39 (40.6%)	33 (34.4%)	
Race				0.094
White	161 (83.9%)	85 (88.5%)	76 (79.2%)	
Black	20 (10.4%)	5 (5.2%)	15 (15.6%)	
Others	8 (4.2%)	4 (4.2%)	4 (4.2%)	
Unknown	3 (1.5%)	2 (2.1%)	1 (1.0%)	
Histological type				1.000
Ductal	183 (95.3%)	91 (94.8%)	92 (95.8%)	
Lobular	9 (4.7%)	5 (5.2%)	4 (4.2%)	
Grade				0.934
1	7 (3.6%)	4 (4.2%)	3 (3.1%)	
2	52 (27.1%)	25 (26.0%)	27 (28.1%)	
3	55 (28.7%)	29 (30.2%)	26 (27.1%)	
Unknown	78 (40.6%)	38 (39.6%)	40 (41.7%)	
Clinical T stage				0.660
1	79 (41.1%)	38 (39.6%)	41 (42.7%)	
2	113 (58.2%)	58 (60.4%)	55 (57.3%)	
ER‐receptor status				1.000
Positive	187 (97.4%)	94 (97.9%)	93 (96.9%)	
Negative	5 (2.6%)	2 (2.1%)	3 (3.1%)	
PR‐receptor status				1.000
Positive	172 (89.6%)	86 (89.6%)	86 (89.6%)	
Negative	20 (10.4%)	10 (10.4%)	10 (10.4%)	
HER2‐receptor status				0.365
Positive	22 (11.5%)	13 (13.5%)	9 (9.4%)	
Negative	170 (88.5%)	83 (86.5%)	87 (90.6%)	
Breast surgery				1.000
Mastectomy	178 (92.7%)	89 (92.7%)	89 (92.7%)	
BCS	14 (7.3%)	7 (7.3%)	7 (7.3%)	
Number of positive lymph nodes				0.645
1	129 (67.2%)	63 (65.6%)	66 (68.8%)	
2	63 (32.8%)	33 (34.4%)	30 (31.2%)	
Sentinel lymph node met size				0.824
Micrometastases (2 mm)	23 (12.0%)	12 (12.5%)	11 (11.5%)	
Macrometastases (>2 mm)	169 (88.0%)	84 (87.5%)	85 (88.5%)	
Chemotherapy				0.764
Yes	122 (63.5%)	62 (64.6%)	60 (62.5%)	
No/unknown	70 (36.5%)	34 (35.4%)	36 (37.5%)	

Abbreviations: ALND, axillary lymph node dissection; BCS, breast‐conserving therapy; HER2, human epidermal growth factor receptor 2; HR, hormone receptor; PR, progesterone receptor; SD, standard deviation; SLNB, sentinel lymph node biopsy.

### 3.5 Sensitivity analysis

A sensitivity analysis, adjusting the definition of SLNB to include dissections of 1–4 lymph nodes, showed no significant differences in OS and BCSS between the groups, both before and after propensity score matching (Table 4).

**TABLE 4:** Comparison of adjusted hazard ratios for overall and breast cancer specific survival in the ALND group versus the SLNB group.

Criteria for SLNB group	Total	Overall survival		Breast cancer specific survival	
		HR (95% CI)	*p*‐value	HR (95% CI)	*p*‐value
ALN ≤ 1					
Unadjusted	168	0.732–4.604	0.195	0.202–4.695	0.975
Adjusted (PSM)	66	0.408–5.143	0.566	0.042–5.070	0.526
ALN ≤ 2					
Unadjusted	210	0.947–4.191	0.069	0.198–2.968	0.700
Adjusted (PSM)	112	0.357–2.904	0.973	0.029–2.311	0.226
ALN ≤ 3					
Unadjusted	255	0.711–2.964	0.307	0.179–2.088	0.432
Adjusted (PSM)	142	0.485–3.114	0.664	0.090–2.671	0.409
ALN ≤ 4					
Unadjusted	281	0.702–2.800	0.339	0.147–1.721	0.274
Adjusted (PSM)	172	0.539–3.724	0.479	0.027–2.175	0.206
ALN ≤ 5					
Unadjusted	299	0.707–2.756	0.337	0.134–1.568	0.214
Adjusted (PSM)	192	0.451–2.402	0.925	0.623–15.307	0.167

Abbreviations: ALND, axillary lymph node dissection; ALN, axillary lymph node; CI, confidence interval; HR hazard ratio; PSM, propensity score matching; SLNB; sentinel lymph node biopsy.

## 4 DISCUSSION

Our investigation has revealed an increasing trend in the adoption of SLNB among early‐stage MBC patients with 1–2 positive sentinel lymph nodes, despite the absence of direct evidence supporting this practice or explicit recommendations in current clinical guidelines. We observed a significant rise in the utilization of SLNB over the study period, suggesting a shift toward less invasive axillary management strategies in this patient population. Crucially, our analysis indicates that the choice between SLNB and ALND does not result in significant differences in key survival outcomes, including OS and BCSS. This finding has profound implications for the axillary management of early‐stage MBC patients who receive adjuvant radiotherapy and adequate systemic treatment, affirming that SLNB can serve as an effective, complication‐reducing alternative without compromising survival.

The biological profile of MBC distinctly differs from that of female breast cancer,^13^ with invasive ductal carcinoma being the predominant histological type and lobular carcinoma relatively rare.^14^ In our cohort, invasive ductal carcinoma accounted for 97.0% of cases, while lobular carcinoma represented only 3.0%. Furthermore, we noted a high prevalence of ER positivity (98.0%) and PR positivity (90.6%), with only a 10.4% rate of Her‐2 overexpression. These findings align with previous reports,^15^, ^16^, ^17^ emphasizing the hormonal receptor positivity and lower likelihood of Her‐2 overexpression in MBC, which may influence treatment responses and prognostic outcomes.

Our analysis reveals demographic nuances within the MBC population undergoing SLNB, notably that patients undergoing SLNB tend to be older (median age 65.9 years) compared with those undergoing ALND (median age 63.1 years), with a statistically significant age difference (*p* = 0.027). This age disparity may reflect a clinical preference for less invasive procedures among older male patients, who are potentially more susceptible to comorbidities. Supporting this hypothesis, data from another national database^9^ indicate a higher prevalence of significant comorbidities (Charlson comorbidity score ≥2) among patients in the SLNB group than in the ALND group (11.2% vs. 6.2%, *p* = 0.0126).

Our analysis uncovered a notable difference in the choice of surgical treatment between the SLNB and ALND groups, with a significantly higher proportion of patients undergoing breast‐conserving surgery (BCS) in the SLNB group (20.8%) than in the ALND group (6.3%, *p* < 0.001). Traditionally, mastectomy has been the predominant surgical option for MBC,^15^ largely due to the typically small volume of male breast tissue and the central location of most tumors, which may render BCS more challenging or cosmetically less satisfactory. However, recent trends indicate an increasing acceptance of BCS in the management of MBC, with the incidence of BCS usage reported to range from 4% to 19.8% in various studies.^12^, ^15^, ^18^ This shift is supported by a growing body of evidence^19^, ^20^, ^21^ suggesting that BCS offers survival outcomes comparable to mastectomy in male patients, a stance now endorsed by the National Comprehensive Cancer Network (NCCN) Breast Cancer Guidelines^10^ for eligible male patients.

Studies^22^, ^23^ have shown that axillary lymph node involvement is more prevalent in MBC, with incidence increasing with age. Men are typically diagnosed approximately five years later than women,^24^ likely due to lower disease awareness and the absence of routine screening protocols for men.^25^ In FBC patients with early‐stage disease and limited sentinel lymph node involvement, opting for radiation therapy over ALND is a safe practice. Our study extends this observation to male patients, highlighting a similar trend toward sparing ALND in cases of limited sentinel lymph node metastasis.

Our study addresses a pivotal question in the treatment of early‐stage MBC with limited sentinel lymph node metastasis: the comparative efficacy of SLNB versus ALND on survival outcomes. While Chung et al.^9^ reported a superior 5‐year OS rate for ALND (83.8%) compared with SLNB (76.0%, *p* = 0.0104), suggesting caution in applying findings from the ACOSOG Z0011 and EORTC AMAROS trials directly to MBC, it is crucial to consider that half of the patients in their study did not receive postoperative radiotherapy. This omission contrasts with the protocols of these landmark trials, where adjuvant radiotherapy plays a critical role in treatment outcomes.

Emerging research^26^, ^27^ underscores the survival benefits of postoperative radiotherapy for patients with lymph node‐positive breast cancer, irrespective of the surgical approach to the breast or axilla. Meta‐analysis by Lin et al.^21^ and a comprehensive review of 1933 MBC patients in the SEER database by Abrams et al.^27^ both highlight the significant improvement in survival rates conferred by adjuvant radiotherapy, particularly for patients with lymph node metastases following mastectomy. This evidence aligns with ASCO guidelines^28^ advocating for radiotherapy in all lymph node‐positive MBC cases.

In our analysis, which excluded patients who did not receive radiotherapy to more accurately reflect contemporary clinical practice, we found no significant difference in 5‐year OS or BCSS between the SLNB and ALND groups (SLNB 77.98% vs. ALND 85.85%, *p* = 0.337 for OS; SLNB 91.54% vs. ALND 94.97%, *p* = 0.214 for BCSS). Even after employing propensity score matching to mitigate confounding factors, we observed no survival advantage for ALND over SLNB (5‐year OS: SLNB 83.9% vs. ALND 82.0%, *p* = 0.925; 5‐year BCSS: SLNB 90.1% vs. ALND 96.9%, *p* = 0.167).

Our categorization of the SLNB and ALND groups, based on the number of lymph nodes removed, considered the reported non‐sentinel lymph node metastasis rates in female patients, which could suggest a higher pathological lymph node stage in our SLNB group. However, adjusting our definition of the SLNB group to account for clinical practice variations in the number of sentinel lymph nodes removed did not reveal a survival benefit for ALND over SLNB, whether in terms of OS or BCSS.

Our investigation marks a pioneering step in addressing the management of axillary lymph nodes in early‐stage MBC patients with positive sentinel lymph nodes. While direct evidence is scarce, our findings suggest a shift toward sparing ALND in cases of limited sentinel lymph node metastasis in the post‐Z0011 era, mirroring trends observed in FBC patients. This trend not only fills a critical knowledge gap in the axillary management of male patients but also provides a compelling rationale for considering less invasive treatment options that could potentially improve the quality of life for MBC patients.

Despite its contributions, our study faces several limitations primarily related to its reliance on the SEER database. The retrospective nature of our analysis and the inherent constraints of the database may leave some confounding factors unaddressed, despite our efforts to mitigate bias through propensity score matching. The lack of detailed records on axillary surgery necessitated a definition of SLNB and ALND based on the number of lymph nodes removed, introducing a potential for selection bias. Additionally, the absence of information on familial genetic susceptibility, particularly BRCA mutations,^29^ and detailed data on adjuvant therapies such as hormonal therapy poses challenges,^30^ given their significance in patient prognosis and treatment outcomes. In our study, we included only patients who received postoperative radiotherapy. However, the SEER database does not provide detailed information on radiotherapy regimens, such as the specific area treated or the dose administered. Given these limitations, we can only assume that patients received guideline‐concordant radiotherapy, which may introduce bias.

The rarity of MBC complicates the recruitment of a sufficient number of patients for clinical analysis, highlighting the difficulties of conducting large‐scale randomized trials. Nonetheless, prospective cohort studies, though challenging, may offer a viable path forward, requiring extensive international collaboration and dedicated research efforts.

## 5 CONCLUSIONS

Our study found no significant survival difference between SLNB alone and ALND in early‐stage MBC with limited sentinel lymph node metastasis who received postoperative radiotherapy. Despite limited direct evidence, the use of SLNB has increased. Our analysis provides preliminary data to support sparing ALND in male patients with cT1‐2 staging and 1–2 sentinel lymph node metastases, informing future guidelines to reduce surgical complications. Large‐scale trials are needed but challenging due to the rarity of MBC.

## AUTHOR CONTRIBUTIONS

**Hanzhao Yang:** Conceptualization (equal); data curation (equal); formal analysis (equal); methodology (equal); writing – original draft (lead). **Yuxia Ruan:** Conceptualization (equal); data curation (equal); formal analysis (equal); writing – review and editing (equal). **Jiabin Wang:** Conceptualization (equal); writing – review and editing (equal). **Jianghua Qiao:** Conceptualization (equal); writing – review and editing (equal). **Zhenzhen Liu:** Conceptualization (equal); methodology (equal); project administration (lead); writing – review and editing (equal).

## FUNDING INFORMATION

This study received no specific grant from any funding agency in the public, commercial, or not‐for‐profit sectors.

## CONFLICT OF INTEREST STATEMENT

The authors declare that there is no conflict of interest.

## ETHICS STATEMENT

As our study analyzed publicly available, de‐identified data from the SEER database, The Institutional Review Board of The Affiliated Cancer Hospital of Zhengzhou University & Henan Cancer Hospital has confirmed that no ethics approval is required for this study.

## Data Availability

The datasets used and/or analyzed during this study are available from the corresponding author upon reasonable request.

## References

[1] Giordano SH . Breast cancer in men. N Engl J Med. 2018;378(24):2311‐2320. 29897847 10.1056/NEJMra1707939

[2] Corrigan KL , Mainwaring W , Miller AB , et al. Exclusion of men from randomized phase III breast cancer clinical trials. Oncologist. 2020;25(6):e990‐e992. 32272505 10.1634/theoncologist.2019-0871 PMC7288651

[3] Pilger TL , Francisco DF , Candido Dos Reis FJ . Effect of sentinel lymph node biopsy on upper limb function in women with early breast cancer: a systematic review of clinical trials. Eur J Surg Oncol. 2021;47(7):1497‐1506. 33549375 10.1016/j.ejso.2021.01.024

[4] McLaughlin SA , Wright MJ , Morris KT , et al. Prevalence of lymphedema in women with breast cancer 5 years after sentinel lymph node biopsy or axillary dissection: objective measurements. J Clin Oncol. 2008;26(32):5213‐5219. 18838709 10.1200/JCO.2008.16.3725 PMC2652091

[5] Giuliano AE , Ballman KV , McCall L , et al. Effect of axillary dissection vs no axillary dissection on 10‐year overall survival among women with invasive breast cancer and sentinel node metastasis: the ACOSOG Z0011 (Alliance) randomized clinical trial. JAMA. 2017;318(10):918‐926. 28898379 10.1001/jama.2017.11470 PMC5672806

[6] Donker M , van Tienhoven G , Straver ME , et al. Radiotherapy or surgery of the axilla after a positive sentinel node in breast cancer (EORTC 10981‐22023 AMAROS): a randomised, multicentre, open‐label, phase 3 non‐inferiority trial. Lancet Oncol. 2014;15(12):1303‐1310. 25439688 10.1016/S1470-2045(14)70460-7 PMC4291166

[7] Raber BM , Lin H , Shen Y , Shaitelman SF , Bedrosian I . Trends in regional nodal management of breast cancer patients with low nodal burden. Ann Surg Oncol. 2019;26(13):4346‐4354. 31605340 10.1245/s10434-019-07901-y

[8] Poodt IGM , Spronk PER , Vugts G , et al. Trends on axillary surgery in nondistant metastatic breast cancer patients treated between 2011 and 2015: a Dutch population‐based study in the ACOSOG‐Z0011 and AMAROS era. Ann Surg. 2018;268(6):1084‐1090. 28742702 10.1097/SLA.0000000000002440

[9] Chung SH , de Geus SWL , Shewmaker G , et al. Axillary lymph node dissection is associated with improved survival among men with invasive breast cancer and sentinel node metastasis. Ann Surg Oncol. 2023;30(9):5610‐5618. 37204557 10.1245/s10434-023-13475-7

[10] Gradishar WJ , Moran MS , Abraham J , et al. Breast cancer, version 3.2022, NCCN clinical practice guidelines in oncology. J Natl Compr Cancer Netw. 2022;20(6):691‐722. 10.6004/jnccn.2022.0030 35714673

[11] Singh R , Cao L , Sarode AL , Kharouta M , Shenk R , Miller ME . Trends in surgery and survival for T1‐T2 male breast cancer: a study from the National Cancer Database. Am J Surg. 2023;225(1):75‐83. 36208958 10.1016/j.amjsurg.2022.09.043

[12] Bateni SB , Davidson AJ , Arora M , et al. Is breast‐conserving therapy appropriate for male breast cancer patients? A national cancer database analysis. Ann Surg Oncol. 2019;26(7):2144‐2153. 30761438 10.1245/s10434-019-07159-4 PMC6545266

[13] Chavez‐Macgregor M , Clarke CA , Lichtensztajn D , Hortobagyi GN , Giordano SH . Male breast cancer according to tumor subtype and race: a population‐based study. Cancer. 2013;119(9):1611‐1617. 23341341 10.1002/cncr.27905 PMC3971835

[14] Vermeulen MA , Slaets L , Cardoso F , et al. Pathological characterisation of male breast cancer: results of the EORTC 10085/TBCRC/BIG/NABCG International Male Breast Cancer Program. Eur J Cancer. 2017;82:219‐227. 28292559 10.1016/j.ejca.2017.01.034

[15] Cardoso F , Bartlett JMS , Slaets L , et al. Characterization of male breast cancer: results of the EORTC 10085/TBCRC/BIG/NABCG International Male Breast Cancer Program. Ann Oncol. 2018;29(2):405‐417. 29092024 10.1093/annonc/mdx651 PMC5834077

[16] Piscuoglio S , Ng CK , Murray MP , et al. The genomic landscape of male breast cancers. Clin Cancer Res. 2016;22(16):4045‐4056. 26960396 10.1158/1078-0432.CCR-15-2840 PMC4987160

[17] Sabih QA , Young J , Takabe K . Management of male breast cancer: the journey so far and future directions. World J Oncol. 2021;12(6):206‐213. 35059080 10.14740/wjon1418 PMC8734504

[18] Elmi M , Sequeira S , Azin A , Elnahas A , McCready DR , Cil TD . Evolving surgical treatment decisions for male breast cancer: an analysis of the National Surgical Quality Improvement Program (NSQIP) database. Breast Cancer Res Treat. 2018;171(2):427‐434. 29808286 10.1007/s10549-018-4830-y

[19] Leone JP , Leone J , Zwenger AO , Iturbe J , Leone BA , Vallejo CT . Locoregional treatment and overall survival of men with T1a,b,cN0M0 breast cancer: a population‐based study. Eur J Cancer. 2017;71:7‐14. 27940356 10.1016/j.ejca.2016.10.038

[20] Zaenger D , Rabatic BM , Dasher B , Mourad WF . Is breast conserving therapy a safe modality for early‐stage male breast cancer? Clin Breast Cancer. 2016;16(2):101‐104. 26718092 10.1016/j.clbc.2015.11.005

[21] Lin AP , Huang TW , Tam KW . Treatment of male breast cancer: meta‐analysis of real‐world evidence. Br J Surg. 2021;108(9):1034‐1042. 34476472 10.1093/bjs/znab279

[22] Yadav S , Karam D , Bin Riaz I , et al. Male breast cancer in the United States: treatment patterns and prognostic factors in the 21st century. Cancer. 2020;126(1):26‐36. 31588557 10.1002/cncr.32472 PMC7668385

[23] Greif JM , Pezzi CM , Klimberg VS , Bailey L , Zuraek M . Gender differences in breast cancer: analysis of 13,000 breast cancers in men from the National Cancer Data Base. Ann Surg Oncol. 2012;19(10):3199‐3204. 22766989 10.1245/s10434-012-2479-z

[24] Giordano SH , Cohen DS , Buzdar AU , Perkins G , Hortobagyi GN . Breast carcinoma in men: a population‐based study. Cancer. 2004;101(1):51‐57. 15221988 10.1002/cncr.20312

[25] Midding E , Halbach SM , Kowalski C , Weber R , Wurstlein R , Ernstmann N . Men with a "woman's disease": stigmatization of male breast cancer patients—a mixed methods analysis. Am J Mens Health. 2018;12(6):2194‐2207. 30222029 10.1177/1557988318799025 PMC6199445

[26] Madden NA , Macdonald OK , Call JA , Schomas DA , Lee CM , Patel S . Radiotherapy and male breast cancer: a population‐based registry analysis. Am J Clin Oncol. 2016;39(5):458‐462. 24781343 10.1097/COC.0000000000000078

[27] Abrams MJ , Koffer PP , Wazer DE , Hepel JT . Postmastectomy radiation therapy is associated with improved survival in node‐positive male breast cancer: a population analysis. Int J Radiat Oncol Biol Phys. 2017;98(2):384‐391. 28463158 10.1016/j.ijrobp.2017.02.007

[28] Hassett MJ , Somerfield MR , Giordano SH . Management of male breast cancer: ASCO guideline summary. JCO Oncol Pract. 2020;16(8):e839‐e843. 32091951 10.1200/JOP.19.00792

[29] Ding YC , Steele L , Kuan CJ , Greilac S , Neuhausen SL . Mutations in BRCA2 and PALB2 in male breast cancer cases from the United States. Breast Cancer Res Treat. 2011;126(3):771‐778. 20927582 10.1007/s10549-010-1195-2 PMC3059396

[30] Venigalla S , Carmona R , Guttmann DM , et al. Use and effectiveness of adjuvant endocrine therapy for hormone receptor‐positive breast cancer in men. JAMA Oncol. 2018;4(10):e181114. 29800030 10.1001/jamaoncol.2018.1114 PMC6233775

